# Sex Differences in Airway Remodeling and Inflammation: Clinical and Biological Factors

**DOI:** 10.3389/falgy.2022.875295

**Published:** 2022-04-29

**Authors:** Carolyn Damilola Ekpruke, Patricia Silveyra

**Affiliations:** ^1^Department of Environmental and Occupational Health, Indiana University Bloomington School of Public Health, Bloomington, IN, United States; ^2^Department of Medicine, Indiana University School of Medicine, Indianapolis, IN, United States

**Keywords:** asthma, airway, sex, gender, lung, hormone

## Abstract

Asthma is characterized by an increase in the contraction and inflammation of airway muscles, resulting in airflow obstruction. The prevalence of asthma is lower in females than in males until the start of puberty, and higher in adult women than men. This sex disparity and switch at the onset of puberty has been an object of debate among many researchers. Hence, in this review, we have summarized these observations to pinpoint areas needing more research work and to provide better sex-specific diagnosis and management of asthma. While some researchers have attributed it to the anatomical and physiological differences in the male and female respiratory systems, the influences of hormonal interplay after puberty have also been stressed. Other hormones such as leptin have been linked to the sex differences in asthma in both obese and non-obese patients. Recently, many scientists have also demonstrated the influence of the sex-specific genomic framework as a key player, and others have linked it to environmental, social lifestyle, and occupational exposures. The majority of studies concluded that adult men are less susceptible to developing asthma than women and that women display more severe forms of the disease. Therefore, the understanding of the roles played by sex- and gender-specific factors, and the biological mechanisms involved will help develop novel and more accurate diagnostic and therapeutic plans for sex-specific asthma management.

## Introduction

According to the most recent National Health Interview survey data, current asthma patients represent about 8% of the United States population (1). Asthma is an inflammatory lung disease characterized by an increase in the contraction and inflammation of the airway and related muscles, resulting in airflow obstruction. In adults, the prevalence of asthma in females is higher than that of males (10.7 vs. 6.5% globally, and 9.8 vs. 6.1% in the US, respectively). While asthma prevalence is greater in male children than in females, it reverses at age 13 (which is mostly the onset of puberty) and continues to about age 65–70 years of adulthood (2). In 2019, the global prevalence of asthma was 136 per million in females, and 127 per million in males (3). In addition, females have been consistently shown to have a higher death rate due to asthma than males (1, 4, 5).

Airway remodeling and hyperresponsiveness in asthma have been documented long ago by many researchers to be the anatomical and physiological alterations that occur in the airway of an asthmatic individual. Some of the pro-inflammatory responses that trigger airway remodeling include the infiltration of eosinophils into the airway, recruitment of inflammatory cells, increase in the secretion of interleukins, increase in immunoglobulin E, shedding of epithelial layers, thickening of the subepithelial layer, increase in smooth muscle mass, increased secretion and sizes of mucous secreting cells, changes in blood vessels associated with the airway, and wearing-off of the soft bones of the airway (6–11). In general, males are known to be less prone to certain immunological illnesses compared to females and the role of sex hormones has been highlighted extensively (12–15). In both human and animal studies, clear sex distinctions have been documented in airway remodeling in asthma. The reasons for these differences have not been fully elucidated, but many studies have implicated the role of hormonal, immunological, occupational, and environmental factors (13, 16, 17). Hence, the purpose of this review is to discuss both sex and gender differences in airway remodeling in asthma, using data available from clinical and animal studies. Understanding the mechanisms underlying these disparities will guide the development of novel sex- and gender-specific diagnosis and therapeutic options for the management of asthma.

## Sex Differences in the Structure and Function of the Respiratory System

In clinical studies, researchers have attributed the sex differences in airway remodeling to anatomical and physiological differences in the structure and function of the respiratory system (Table 1). Others argue that the observed differences are majorly due to contributions of sex hormones and other sex-specific biochemical processes. Some of the anatomical observations reported are in the nasal cavity and floor, which is lower in females compared to males (18), and cranial airways which seem smaller in females than males (19). It has also been shown that the upper airway compliance during non-rapid eye movement sleep is lower in females than males though it was observed that the neck circumference and surface area of the body are also involved (20). Some earlier studies also reported observations in the pharynx, which is smaller in size and cross-sectional area and has low resistance in females than in males (21–23). These are areas of the respiratory system that are known to humidify and warm the inspired air, as most of them are lined with columnar epithelial cells that secrete mucus. They also participate in the mucocilliary clearance process when aerosol particles are inhaled, which makes them very important in allergic airway diseases. Overall, while the lung of adult males is bigger than that of females, it is also said to be age and stature-dependent (25). The size of the lungs influences the total lung capacity (TLC) of an individual, as TLC is directly proportional to the size of the lung. TLC also reflects the amount of air that goes in and out of the lungs, which is affected in asthma. Additionally, the number of alveoli is higher in adult males than females. Since the alveoli are the major sites where the exchange of gases occurs in the respiratory system, males having a larger surface area for gaseous exchange when compared to females, can influence asthma risk (25, 30, 31).

**Table 1:** Sex differences in the respiratory system structure and functions.

**Respiratory system structures/functions**	**Females (vs. Males)**	**References**
**Nasal cavity and cranial airway**		
Length	Shorter	(18, 19)
Size	Smaller	
Width	Wider	
Upper airway Compliance	Lower	(20)
**Pharynx**		(21–23)
Size	Smaller	
Cross-sectional area	Smaller	
Resistance	Lower	(24)
**Lung**		
Size	Smaller	(25)
**Alveoli**		
Count	Lower	(26, 27)
Surface area	Smaller	
**Immune cell populations**		
Regulatory T cells	Lower	(28, 29)
CD4+ and CD4+/CD8+ ratio	Higher	

## Sex Differences in Immune Responses in Asthma

Sex dimorphism in immune response has been reported by many researchers both in animal and human studies. Innate and adaptive immune responses were said to be lower in males than in females. Many scientists have attributed the observed difference as being influenced by sex hormones. For instance, in animal studies, the activities of macrophages were lower in males compared to that of females attributing this to the protective role of male sex hormone (32). This report was substantiated with that the finding that antibody and cell-mediated responses were low in males than in females only when their testosterone level was at its peak (33, 34). In animal studies, regulatory T cells have been shown to play vital roles in mechanisms of inflammation in allergy, by preventing the production of cytokines such as TGF-beta and interleukin (IL)-10 (35), and proliferation of T cells (36), though the mechanisms involved are not clearly understood. These cells are very few in the lungs of female mice compared with that of the males (26, 27). Moreover, the subsets of CD4+ and CD8+ T cells were found to be numerous in the peripheral blood and lung tissue of asthmatic patients (37, 38). Inflammation mediated by allergens is said to be dependent on CD4 and CD8 cells rather than the previous belief that solely implicates immunoglobulin E and B cells since the lungs of knockout mice for immunoglobulin E and B still produce an allergic-mediated inflammation with house dust mite sensitization (39). CD8+ T cells are known for the role they play in immune tolerance, and they are a good source of proinflammatory cytokines in asthma (40) CD4+ cells and the ratio of CD4+/CD8+ cells (a marker of chronic lung disease) (41) are lower in males than females throughout adulthood (28, 29). Lamson et al. concluded that female mice express genes associated with adaptive immune response, while male mice express genes associated with innate immunity (42). Combined, these features may contribute to the observed sex differences in airway remodeling in asthma.

## Sex-Differences in the Hormonal Interplay of Airway Remodeling in Asthma

In clinical studies, a strong link has been established between the hypothalamic-gonadal-pituitary axis and the lungs (43). Many physiological functions of the lungs have been linked to the influence of different hormones throughout the life span. For instance, the influence of hormones on physiological surfactant secretion, lung development, and inflammatory markers production and functions have been documented by many researchers (28, 44–49). Estrogen has been named a key player in the quick maturation of surfactant-producing cells; this explains the faster development of female lungs compared with that of males (50–52). The production of surfactants also decreases as estrogen levels decline in females with an increase in age. On the other hand, androgens have an inhibitory effect on the production of surfactants at a young age (53). The role of surfactants has also been suggested in the recruitment of inflammatory cells in asthma (54). Similarly, a strong correlation between the production of surfactant and eosinophil counts has been shown, suggesting that it may serve as an immunomodulator (54).

Estrogen and its receptors (alpha and beta) have been demonstrated in human studies to play a vital role in the regulation of anatomical and physiological functions of the airway (55–59), as reviewed by us in (60). However, the role of estrogen and its receptors in airway remodeling in asthma has been controversial (61). Earlier animal studies reported suppression of the immune system with the increase in estrogen levels in the circulatory system (62). Hormonal fluctuations in the mouse estrous cycle influenced the expression of inflammatory genes in ozone-challenged female mice (63). Estrogen was also found to increase airway inflammation by enhancing the activities of the T-helper cells type 2 in asthma (14). Thus, it has been suggested that the role of estrogen can be both destructive and advantageous depending on whether the alpha or the beta estrogen receptor signaling pathway is in use.

The nuclear estrogen receptors alpha and beta are both found in the respiratory system but different proportions (62). One study reported that the activation of the beta estrogen receptor showed a reduction of extracellular matrix in asthmatic humans by suppressing the activity of the NF-kB pathway (58). Another study showed that beta estrogen receptor activation led to the proliferation of airway smooth muscle cells by inhibiting the activities of the platelet cells (55). The same study showed that estrogen inhibits smooth muscle contraction by reducing calcium ion influx during inflammation in asthmatic conditions (57).

Another female hormone, progesterone, and its receptors (alpha and beta receptors) have been implicated in sex differences in asthma (16). This hormone is present in both males and females, but the level is higher in females than in males (64). Many decades ago, progesterone was found to enhance the dilation of bronchi (65–67). However, there is little information available for the role it plays in the allergic immune response. Some researchers have documented that progesterone contributes to the effects of other sex hormones. For instance, it is known to have a high affinity for the enzyme 5-alpha reductase that helps in the conversion of testosterone to active 5-dihydrotestosterone (64). Other studies have shown that testosterone is inactive in the presence of progesterone (68). In postmenopausal women undergoing hormonal therapy, the combination of progesterone and estrogen increases their risk of developing asthma, though the associated mechanisms have not been studied (69). A positive correlation of serum progesterone and peak flow rate in different menstrual phases was also found (70). Also, progesterone was said to regulate the production and activities of IL-17 which was enhanced in women with severe asthma (71). Similarly, human airway epithelial cells treated with progesterone display a reduced frequency of cilia movement, indicating that progesterone negatively affects the functions of the micro ciliary apparatus (72). Since the levels of this hormone are higher in females than in males, it is possible that progesterone fluctuations in females contribute to asthma susceptibility.

Regarding male hormones, testosterone and its associated metabolites also play a significant role in modulating T cell activity, which helps provide an equilibrium between hypersensitivity reactions and the body's defense system. Testosterone is classified as an immune-protective hormone, along with 5-alpha dihydrotestosterone (5-alpha DHT). Both help reduce airway inflammation in asthma by reducing the response of the innate and adaptive immunity (14).

Adipose tissue is known for its ability to store energy in form of triglycerides, recently, it becomes a recognized endocrine organ. It is known to secrete hormones such as adiponectin, C1a-TNF related protein 9, retinol-binding protein 4, leptin, and omentin (73, 74). This group of hormones is referred to as the adipokines in addition to the proinflammatory and anti-inflammatory cytokines secreted by the same tissue. All these go into the circulatory system where they mediate the activities between adipose tissue and other tissues/ organs of the body (75). The ability of the adiponectin to produce more than one effect makes it an interesting adipokine to study among researchers. It is a general belief that it has an anti-inflammatory property. This was demonstrated in the research of (76), though it was not in lung disease cardiovascular system disease. Adiponectin is known to carry out its anti-inflammatory effect by acting on the macrophages through the prevention of progenitor myeloid cells differentiation (77, 78). It also possesses the ability to alter the activities of the macrophages and toll-like receptor 4 (79). The role of some adipokines in pulmonary diseases has been reviewed by (80).

Leptin is a hormone of great interest in airway remodeling in asthma, as its levels are known to be enhanced during allergic reactions (81). A few studies tried to implicate this hormone as a key player in the strong association existing between obesity and asthma. However, there are very few studies on the roles of this hormone and its mechanisms of action. There is a great expression of leptin receptors (alpha and beta) in bronchoalveolar epithelial cells and alveolar macrophages (82–84), as well as in other immune cells (85, 86). Decades ago, leptin was shown to stimulate the release of IL-6 and tumor necrosis factors from adipose tissue cells (87, 88). Interleukin 6 is known for its role in the release of the inflammatory marker, C-reactive protein, from the liver in inflammatory conditions (89, 90), as well as in mediating interferon production in T helper 1 cells (91, 92). In mice, the level of macrophage inflammatory protein-2 was directly proportional to the serum level of leptin in ozone-induced airway inflammation (93). Leptin has also been found associated with lung injury and asthma (94). The levels of leptin were found to be higher in children with asthma compared with healthy controls (95). In the same study, the non-asthma group displayed sex differences in leptin levels, whereas no sex differences were observed in the asthma group. In mouse studies, leptin administered exogenously led to an increase in secretions of tumor necrosis factor, IL-6, and IL-12 (95).

Multiple researchers have demonstrated the role of hormones and their various receptors in airway remodeling in asthma using experimental animals. Of great interest, the role of estrogen and its receptors, alpha, and beta, have been documented. The importance of both receptors was seen in lung maturation and the size of alveoli, but the beta estrogen receptor was found to contribute to a larger extent than the alpha in lung elasticity. The effect was more pronounced in male vs. female mice knockout for the beta estrogen receptor (49). In addition, gonadectomized ovalbumin-asthma-induced mice showed a significant increase in infiltration of eosinophils, lymphocytes, and some interleukins in their airway compared to control mice. Dimitropolou et al. also demonstrated the role of estrogen in mouse isolated trachea rings sensitized with serum from asthma and healthy patient (96). Contraction occurred in the trachea ring sensitized by asthmatic serum when carbachol was added but when the same sample was pretreated with estrogen, the contraction was abolished. The authors concluded that the estrogen beta receptor was activated, decreasing the contraction through the stimulation of potassium channels. Other researchers reported sex differences in airway remodeling and attributed the effects to sex hormones. In one study by Riffo-Vasquez et al. female mice that have undergone ovariectomy before being sensitized with ovalbumin showed a reduction in IL-5 levels, eosinophil infiltration, and hyperresponsiveness to methacholine compared to control mice (97). Similarly, Takeda et al. reported a reduction in eosinophil counts, production of serum IgE and hypersensitivity of the airway in ovariectomized mice challenged with ovalbumin or house dust mites (98). Also, in ovalbumin-sensitized females, an increase in airway hyperresponsiveness was observed with no effect on inflammation of the airway after the activation of the alpha estrogen receptor (99).

The role of progesterone and its receptors on airway remodeling in asthma has not been widely studied in experimental animals. Administration of progesterone contraceptives to females in an influenza A mouse model helped to enhance lung function and the repair of the damaged epithelium caused by the infection and inflammation (100). In contrast, Hellings et al. rather reported a worsened airway disease in mice following the exogenous administration of progesterone as it led to an increase in infiltration of eosinophils to the airway and hyperresponsiveness of the airway (101). In type 2 helper cell-mediated immune responses, progesterone stimulates the production of some proinflammatory cytokines (102). This study also confirmed that the female sex hormones play important roles in airway remodeling in asthma.

Male sex hormones have been also documented to play a role in inflammation of the airway mediated by type 2 cells in animal studies. A study by Yu et al. investigated the role of male sex hormones and their derivatives on airway remodeling in asthma (103). By adding DHEA (dehydroepiandrosterone) to the diet of house dust mite sensitized mice, they observed a significant increase in the resulting airway inflammation and infiltration of eosinophils and interleukins into the airway compared to mice eating normal chow, though there was no change in the immunoglobulin E level of both groups (103). The study did not consider sex as a biological variable.

## Sex Differences in Biomarkers of Type 2 Inflammation in Asthma

Sex differences in biomarkers of type 2 inflammation expressed in asthma have been widely investigated (Table 2). Clinical studies have reported sex differences in asthma control by measuring such inflammatory biomarkers, blood eosinophils, exhaled nitric oxide, and serum E levels, and indicated a significantly high symptom control in males compared to the females in the same age group (104, 110). Other researchers found no differences in C-reactive protein in asthma (108). In animal studies of ovalbumin-induced asthma, infiltration of eosinophils, as well as the concentration of serum immunoglobulin E (IgE) and IL-3., were found to be increased in the lungs of female mice when compared to males (98, 111). Similarly, in studies where sex differences in airway-remodeling were hypothesized, the serum concentration of IgE was found to be increased in the female vs. male lungs in two different asthma models used (ovalbumin challenge and house mite dust exposure) (109). The responsiveness of the airway to methacholine was also higher in females than in males. Card et al. also observed that male mice displayed higher airway hyperresponsiveness during methacholine challenge than females (112). This study is one of very few whose observations reflect that male lungs are more affected by asthma than females. The studies of Melgert et al. (26) and Okuyama et al. (113) support the fact that females are more susceptible to airway inflammation caused by ovalbumin challenge than males (26, 113). Both studies observed an increase in airway hyperresponsiveness, eosinophil, T, and B cell counts, and level of cytokines in female vs. male mice challenged with ovalbumin. Moreover, Treg cells (known for their vital role in the prevention of inflammation in allergy) were found lower levels in the lungs of female mice compared to males (26).

**Table 2:** Sex-difference in biomarkers of type 2 inflammation: comparing male and female responses.

**Type 2 Inflammatory markers**	**Responses in Females (vs. Males)**	**References**
Infiltration of eosinophils	Increased	(26, 104) (105)
Expression of interleukins	Increased	(105–107)
Group 2 innate lymphoid cell count	Increased	(14)
T-helper cells (type 2)	Increased	(55)
Extracellular matrix	Decreased	(55)
Platelet cells activities	Decreased	(57)
Smooth Muscle Contraction	Decreased	(43)
Expression of inflammation genes	Increased	(72)
Micro ciliary apparatus activity	Decreased	(104)
Exhaled Nitric oxide	Increased	(104)
Serum Immunoglobulin E levels	Increased	(108)
C-reactive Protein	No significant difference	(26, 109)
Responsiveness of the lugs to methacholine	Increased	(26)

The recently discovered type 2 innate lymphoid cells (ILCs) have been reported to increase in peripheral blood (114, 115) and sputum (116) of patients with asthma. They are known to mediate allergy responses in the lungs (117–119). This was further substantiated by a study reporting an increase in circulating type 2 ILCs counts in women compared with men in a population with moderate to severe asthma (105), where 5-alpha DHT decreased these cell counts and expression of IL-5 and IL-13, both involved airway inflammation and hyperresponsiveness (117). These type 2 ILCs have an inflammatory subset that is found in the lungs and can move in the different mucous-producing sites during airway remodeling in asthma (120). One of the major roles of these cells is the expression of GATA binding protein 3 and inflammatory cytokines. In the lungs, the transformation of precursor ILCs to type 2 ILCs depends on the GATA binding protein 3. Interestingly, these cells are found to be more numerous in females than males (121). The inflammatory subset of type 2 ILCs activates the lectin receptor G1 (116), which is also higher in females. This special subset of type 2 ILCs is known for its role in cytokine production, and it also increases in number with age. To explain the sex difference observed, studies in gonadectomized and estrogen alpha knock-out mice showed that male sex hormones, but not female hormones, regulate the proliferation and function of type 2 ILCs (121). This agrees with the findings of Warren et al. who observed an increase in IL-33 production by type 2 ILCs in female vs. male mice sensitized with ovalbumin (106). Moreover, Laffont et al. showed that the androgen receptor signaling reduced type 2 ILCs proliferation (122). They also observed a sex disparity in the lymphoid cell counts, which was abolished, and type 2 mediated inflammation restored, after orchiectomy.

## Sex Differences in the Association of Environmental, Occupational, and Social Factors With Asthma

Several epidemiological studies have attributed the sex differences in airway remodeling in asthma to environmental, occupational, and social factors. Hence, it is important to distinguish sex as a biological factor vs. gender as a social construct in these analyses (123). Female gender and tobacco smoke have been greatly associated with severe refractory asthma (124).

Occupational factors have also been implicated in the observed sex differences of airway remodeling in asthma. Gendered roles and changes in occupations traditionally performed by men or women can influence asthma development (125). Recently, females were reported to work in highly polluted places like hospitals, homes, and schools, and thus display a higher frequency of work-related asthma was observed in females than in males (126). Interestingly, women are also known to have more pets at home (127), and are negatively affected by secondary exposure to tobacco smoke (127) than men.

Another factor potentially contributing to sex differences in asthma is exercise (128). While a few studies have documented sex differences in immune cell counts (129–131), plasma cytokine levels (129, 131), and lymphocyte apoptosis (132) with different kinds of exercise, others reported no differences, particularly in treadmill running, bicycle, and strength training (133–135). In women, a few studies have linked immune response changes during exercise to the menstrual cycle. For instance, a study in cyclists observed an alteration in leukocytes and cytokine expression in female cyclists during the menstrual phase (134). Others found that regulation of inflammatory genes depends on the time and duration of exercise during the menstrual phase in females (136). In this context, female athletes were found to exhibit severe exercise-induced bronchoconstriction in the luteal phase of the cycle (137). A potential mechanism for this involves differential expression of pro-inflammatory genes. Northoff et al. found that proinflammatory genes are upregulated in the follicular phase, while anti-inflammatory genes are downregulated in the luteal phase in females compared with males (136). Women in the mid-luteal phase also display worsened lung function and exacerbated bronchoconstriction induced by exercise (138). These changes in the mid-luteal phase were associated with an increase in progesterone levels, although the exact mechanism remains unknown (137).

## Obesity, Body Mass Index, and sex Differences in Asthma

Obesity, body mass index, and serum IgE have been associated with asthma across the life span (139, 140). A study in a mixed population of smokers without any respiratory disorders showed a strong association between fat distribution and normal lung function (141). Other studies have shown sex specific associations, including a strong association between new asthma symptoms and high body mass index only in females (142, 143), and a higher prevalence of obesity in women with vs. without asthma (124, 144). In a study of 4,197 asthma patients from the 2012 National Health Interview Survey, a positive association of obesity and body mass index with asthma (overall and allergic) was found (145). The authors also reported that the association of class III obesity (BMI≥40) and asthma was stronger in women. Similarly, epidemiological studies have shown that asthma occurring with obesity (also known as “obesity-associated asthma) is more difficult to treat, more severe, and more prevalent in women (146–148). Finally, mouse models of asthma have also shown that high fat diet and the consequent weight gain contribute to the progression of allergic asthma with females displaying airway remodeling phenotypes at earlier stages than males (149, 150).

The interaction between genetics and environmental factors has also been documented as a contributing aspect in females with asthma and obesity (127, 151, 152), or males (153, 154). However, some studies have reported that the association between asthma and obesity is not gender-based (155). Though this association has been an issue of debate among many researchers (156–158) there is a need to determine the physiological parameters and hormonal influences on airway remodeling and asthma. As indicated earlier, leptin levels correlate with body mass index (92, 159), and human adipose tissue expresses inflammatory cytokines (87, 160). The mechanism through which obesity is linked to asthma has not been fully understood as very few or no animal studies have been carried out in this area.

## Influence of the Genetic Framework in Sex Differences in Airway Remodeling in Asthma

**Figure 1:**
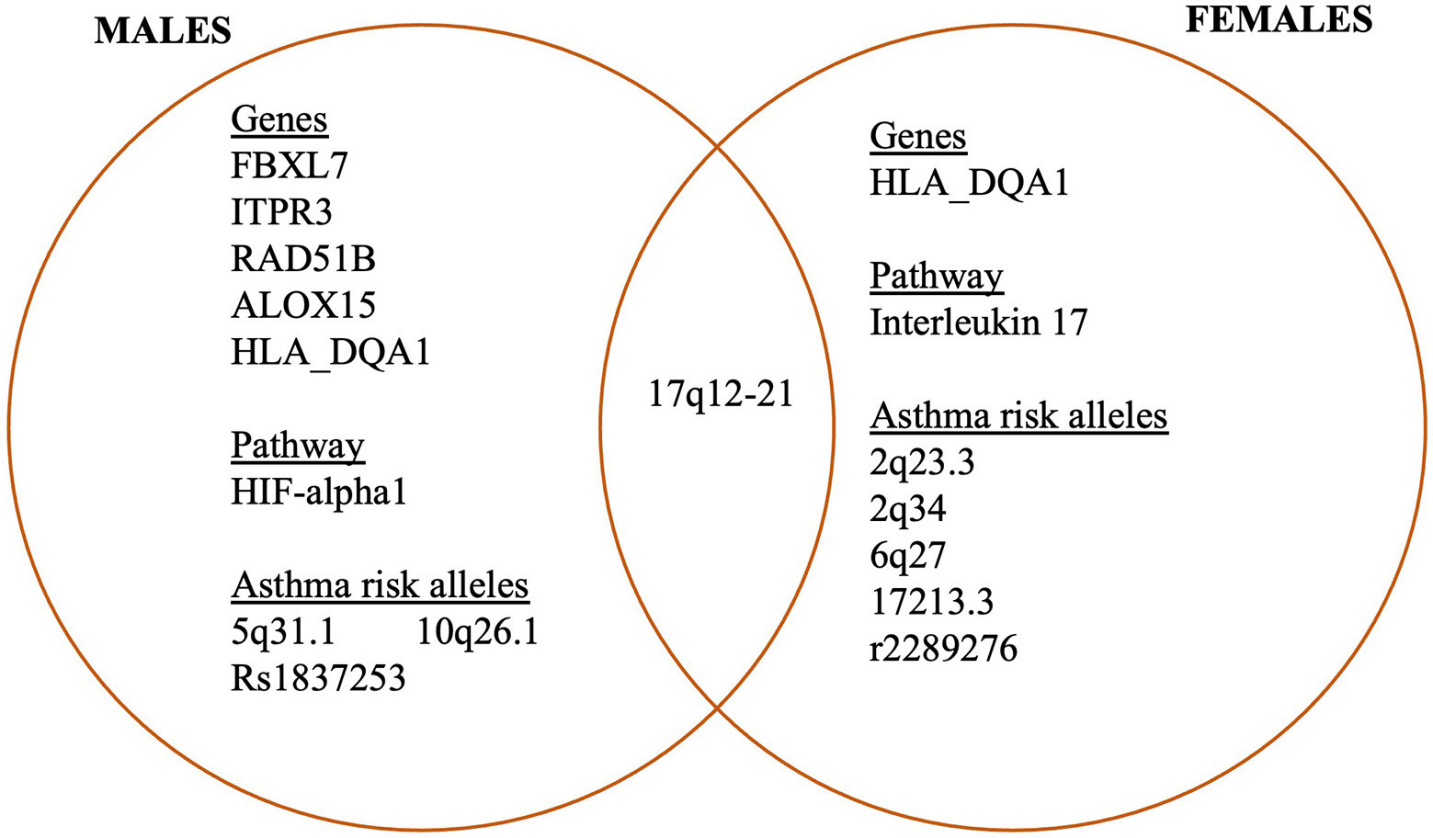
Sex differences in the expression of genes associated with asthma.

There is a paucity of data concerning the influence of genetic framework and pathways on sex differences in airway remodeling in asthma. The genomic framework of males and females with asthma displays numerous differences as described in Figure 1. Gautam et al. identified differentially expressed genes (*n* = 32), as well as both male and female-specific genes (males = 439; females = 299) (161), of which five are impaired in the regulation of physiological processes during asthma. Of these genes, four were male-specific while only one was female-specific (161). They discovered that the majority of these genes were found in the airway epithelium. The four genes specific to the males included *FBXL7, ITPR3, RAD51B, and ALOX15*; three of them were found in the airway epithelial tissue, while only *ALOX15* was found in the blood. All these genes are upregulated in asthma except *RAD51B*. The only gene that was specific to the female is *HLA_DQA1* (downregulated) and also found in the airway epithelial tissue.

Some biological pathways influence the sex difference observed in asthma, and these include *HIF*- alpha 1 and *IL-17* signaling. The *HIF*- alpha 1 signaling pathway has been mostly identified in male-specific differentially expressed genes (161). This pathway is known to play a role in the regulation of pro-inflammatory cytokines, chemokines in processes of infection and allergy. However, the role of this pathway in asthma, and specifically sex differences in airway remodeling has not been studied. Regarding *IL-17* pathways, they have been found upregulated in females with asthma (161–163), but negatively correlated with airway hyperresponsiveness to methacholine (164). This *IL-17* signaling pathway is known for its role in stimulating the cells of the epithelium to produce cytokines that help to recruit neutrophils to the inflammation site (165). Excess production of *IL-17A* has been linked to the risk of developing severe asthma (166–169). This was substantiated by the research of Laan et al. where it was reported that stimulation of the human epithelium with *IL-17A* led to greater expression of *CXCL8 mRNA*, which in turn enhanced neutrophils migration to the inflammation site. Meanwhile, Busse et al. documented that *IL-17A* does not affect the severity of asthma when patients were treated with brodalumab, a human anti-*IL-17* receptor A monoclonal antibody (170).

Many studies focused on sex differences in asthma after adolescence identified that changes in DNA methylation in blood and lung tissue could play a role in the observed disparities (171–178). Furthermore, the interaction between sex-specific and sex-stratified genomes has also been found associated with childhood asthma (179). In this regard, the *17q12-21*-asthma locus was highlighted to be associated with asthma in both males and females but was widely significant in females (179). Similarly, the ligand-dependent nuclear receptor co-repressor-like gene, located within the regulatory region was highlighted in males only (179). This gene peculiar to the males only is known to play a vital role in the determination of height and sperm production.

Asthma risk alleles have also been identified in a genome-wide interaction study. Four (*2q23.3, 2q34, 6q27, and 17213.3*) of these alleles are specific to females, and two (*5q31.1 and 10q26.1*) are specific to males (180). Some single nucleotide polymorphisms (SNPs) that are sex-specific and associated with asthma were discovered in beta 2 adrenergic receptors (181) and thymic stromal lymphopoietin (*TSLP*) (182). The beta 2 adrenergic receptor was observed to have genetic variants that are associated with the development of severe asthma (181). On other hand, *TSLP* is a cytokine similar to IL-17 produced by the cells of the epithelium during an allergic reaction. It is known to be associated with serum IgE in girls. It is also known to play a crucial role in the regulation of allergic responses, specifically, airway inflammation in animal models (183). Recently, this observation was substantiated in *TSLP* knockout mouse studies (184, 185). The specific SNPs discovered in *TSLP* include *rs1837253*, associated with the risk of asthma in males, and *r2289276* in females only (182). Gauderman et al. also identified two other loci, GRIA2 and TNTRSFIIB associated with gene sex and childhood asthma using a genome-wide interaction scan in children exposed to traffic air pollution (186). The influence of sex-interact in the association between interferon-gamma gene, a protein-coding gene, and childhood asthma was also reported (187). This study showed that genotype-sex interactions on asthma were only significant at *rs2069727* and *rs2430561*interferon gamma SNPs though the link was not additive. These genes have shown effects on interferon's response to bacterial infections and developing asthma in early childhood (187). Some researchers have argued that the genotype-sex interaction and asthma were different in each race. For instance, a gene variant known as *KCNMBI* was specifically identified in the African American race and is known to play a role in the contraction of bronchial smooth muscle, hence, influencing the pulmonary function (188). This gene has some other variants, of great interest is the 8,187 allele that was seen to decrease the lung function concerning low FEV_1_% recorded; the damaging effect of the allele was suppressed through an estrogen-mediated upregulation of the high conductance voltage calcium-potassium channel in animal experiments (188).

## The Role of Epigenetic Changes in Sex Differences in Airway Remodeling in Asthma

Epigenetics include changes in the genetic materials that not affect the DNA sequence (189). These changes are characterized by methylation of DNA, modification of histones, and microRNAs (190). Epigenetic changes can be caused by diet, air pollution exposure, tobacco smoking, and drug administration, among other factors, and can occur in any stage of life (190). The external environment is in direct contact with the lungs, hence epigenetic alterations occur in the respiratory tract. Associations of epigenetic changes, including DNA hypermethylation or hypomethylation have been identified with asthma (191–193). The hypomethylated or hypermethylated level in specific genes varies depending on the asthma phenotype. For example, hypermethylation of genes such as *ARG1, ARG2* and *ADAM33* in buccal cells and bronchial epithelial cells have been correlated with asthma phenotypes (194, 195). On the other hand, DNA hypomethylation has been associated with *IL6* and *ADAM33* expression in bronchial fibroblasts and the nasal epithelium (194, 195). As recently reported in a review by Chowdhury et al. certain CpG sites located in the interferon-related developmental regulator 1 (*IFRD1*) gene have been linked to sex-specific effects in asthma (140). However, there are very few studies that discussed the issue of sex differences in asthma associated with epigenetics. One of such research include the Ascaris exposure that was said to lower lung function and increase the risk of asthma development at a higher rate in males than females (196).

## Sex-Differences in miRNA Expression in Asthma asnd Airway Remodeling

**Figure 2:**
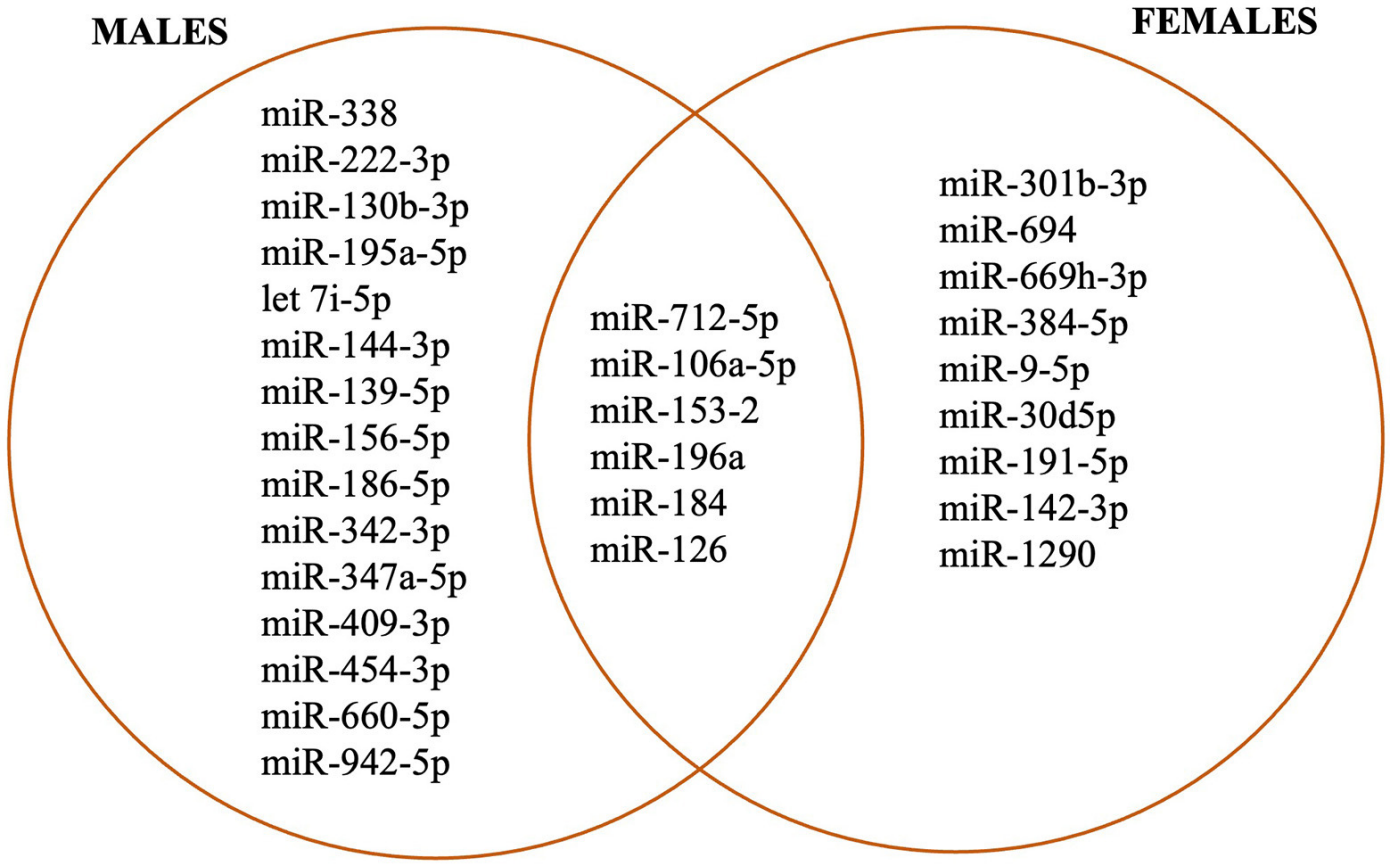
Sex-specific miRNAs associated with airway inflammation and remodeling.

The major characteristics of asthma are airway remodeling with evidence of airway inflammation, increased production of mucus, increased migration of eosinophils to the airway, and airway hypersensitivity. These processes are highly regulated by the expression of inflammatory genes. MiRNAs are a class of small non-coding RNAs that play important roles in gene expression regulation (197). miRNAs are known to be associated with many respiratory disorders including asthma (198, 199) by serving as a biomarker, and mediating interactions among cells (200–202). There is a paucity of data about the sex-specific miRNA expression in asthma. However, immune responses have been known to be sex-specific and miRNAs are said to play a role in this sex bias as shown in Figure 2. Animal studies of ozone-induced lung inflammation reported sex differences in lung miRNA expression (203). In this study, nine sex-specific miRNAs were found in the ozone-induced inflammation group (203). Six of the identified miRNAs were greatly expressed (upregulated) in males (*miR-338, miR-222-3p, miR-130b-3p, let 7i-5p, miR-195a-5p, miR-144-3p*) while five were highly expressed (upregulated) in females (*miR-301b-3p, miR-694, miR-669h-3p, miR-384-5p, miR-9-5p*). Only one was downregulated in females (*miR-30d5p*). They also noted that there were two miRNAs (*miR-712-5p, miR-106a-5p*) that were expressed in both males and females exposed to ozone but with different patterns by sex. They suggested the role of hormones in the pattern of expression. In a study by another group of animals exposed to smoke in *utero*, three miRNAs (*miR-153-2; miR-196a; miR-184*) were identified in both sexes but they were differentially expressed only in males during normal lung development (204). Also, using serum from asthma patients, Kho et al. identified 22 miRNAs that were associated with lung function, nine of which were solely expressed in males (*miR-139-5p; miR-156-5p, miR-186-5p, miR-342-3p; miR-374a-5p, miR-409-3p, miR-454-3p, miR-660-5p; miR-942-5p) while only three were expressed in females (miR-1290; miR-142-3p; miR-191-5p*) (205). In addition, miR-126 was found to be expressed in both sexes (206). Other researchers have also identified miRNAs associated with immune system regulation (207, 208), type 2 immunity (209), regulation of allergic-induced inflammation in asthma (210), and asthma pathogenesis (211). The sex-specific expression of these miRNAs suggests their role in the sex-disparity of airway remodeling in asthma.

## Conclusion

Asthma is a lung inflammatory disease with clear sex differences in incidence, prevalence, and severity across the life span. In both clinical and animal studies, airway remodeling in asthma is known to be characterized by the alterations in the airway leading to obstruction of airflow. The prevalence of asthma suddenly changes at the onset of puberty. Whether this switch is also reflected in airway remodeling has been an issue of debate among researchers over many decades. Many scientists have attributed it to the differences in the structure and functions of the respiratory system, including the nasal cavity, upper airway compliance, lung size, alveoli, and the population of immune system cells in males and females. In great curiosity, many studies linked these sex disparities to changes in sex hormones at puberty, since there is usually a great alteration in the level of these hormones majorly at the onset of puberty. The role of estrogen, testosterone, and progesterone alongside their receptors has been well documented to date. Another hormone implicated in this observation is leptin, although there is still a paucity of data concerning the mechanisms involved and the role it plays in airway remodeling in asthma. The sex differences in biomarkers of type-2-inflammation have also been reported, many of which were suggested to be protective in the male respiratory system, suggesting that females are more susceptible to asthma than males. More recently, researchers have tried to link the observed sex differences in asthma to the genomic frameworks of males and females. This led to the discovery and identification of many sex-specific genes, gene variants, and miRNAs that are directly linked to lung function, lung inflammation, and asthma in general, though much research is still ongoing in this area. Another major concern is the strong link between asthma and each of occupational, environmental, and lifestyle factors that are stronger in females than in males. Many of these observations were from epidemiological studies, thus more experimental studies in this area are highly needed to identify sex-specific mechanisms and pathways involved. However, it is very important to review lessons learned from both clinical and animal studies as this helped us identify the gaps that are needed to be filled to justify the sex difference of airway remodeling in asthma. This will help to intensify efforts in such areas to identify proper sex-specific diagnosis and therapeutic pathways in the management of asthma.

## Author Contributions

CE and PS contributed to the conception of the study and wrote sections of the manuscript. CE organized the database, extracted information, and wrote the first draft of the manuscript. All authors contributed to manuscript revision, read, and approved the submitted version.

## Funding

This study was supported by the National Institutes of Health, R01HL159764 and R03HL141618 (PS).

## Conflict of Interest

The authors declare that the research was conducted in the absence of any commercial or financial relationships that could be construed as a potential conflict of interest.

## Publisher's Note

All claims expressed in this article are solely those of the authors and do not necessarily represent those of their affiliated organizations, or those of the publisher, the editors and the reviewers. Any product that may be evaluated in this article, or claim that may be made by its manufacturer, is not guaranteed or endorsed by the publisher.
